# Anterolateral papillary muscle suction causing low flow in a COVID-19 patient without medical history: a case report of central extracorporeal life support with left ventricular apex decompression

**DOI:** 10.1186/s40981-024-00701-8

**Published:** 2024-04-10

**Authors:** Tomoaki Miyake, Kimito Minami, Masahiro Kazawa, Naoki Tadokoro, Kohei Tonai, Satsuki Fukushima

**Affiliations:** 1 Department of Critical Care Medicine, National Cerebral and Cardiovascular Center, 6-1 Kishibeshinmachi, Suita, Osaka 564-8565 Japan; 2 Department of Anesthesiology, National Cerebral and Cardiovascular Center, 6-1 Kishibeshinmachi, Suita, Osaka 564-8565 Japan; 3 Department of Cardiac Surgery, National Cerebral and Cardiovascular Center, 6-1 Kishibeshinmachi, Suita, Osaka 564-8565 Japan

**Keywords:** Left ventricular decompression, Low flow, Cardiogenic shock, Extracorporeal life support, Transesophageal echocardiography, Papillary muscle suction

## Abstract

**Background:**

Left ventricular (LV) decompression is an essential strategy for improving early survival in patients with refractory cardiogenic shock. Low pump flow in patients on extracorporeal life support (ECLS) with LV apex decompression is a life-threatening issue. However, identifying the underlying causes of low flow can be challenging.

**Case presentation:**

A 38-year-old woman with COVID-19-related fulminant myocarditis was treated with central ECLS with LV apex decompression. The pump flow in the intensive care unit (ICU) was intermittently low, and low flow alerts were frequent. The initial evaluation based on pressure monitor waveforms and transthoracic echocardiography failed to identify the underlying cause. Prompt bedside transesophageal echocardiography (TEE) revealed that the anterolateral papillary muscle was suctioned into the vent cannula of the LV apex during systole. The patient underwent a repeat sternal midline incision in the operating room, and the cannula at the LV apex was repositioned. There were no further suction events after the repositioning, and the patient was weaned from ECLS 12 days after admission to the ICU. The patient was discharged in a stable condition and without neurological deficits.

**Conclusions:**

TEE is an important diagnostic tool to identify the underlying cause of low flow flow in patients undergoing ECLS with LV apex decompression.

**Supplementary Information:**

The online version contains supplementary material available at 10.1186/s40981-024-00701-8.

## Background

Left ventricular (LV) decompression is crucial for managing refractory cardiogenic shock with short-term mechanical circulatory support [1]. It enhances myocardial recovery potential, expedites patient recovery, and improves early survival outcomes [2]. Perioperative management using central extracorporeal life support (ECLS) with LV apex decompression is complicated by intricate patient-device interactions. This report presents a unique case of intermittent low-flow alarms stemming from the suction of the anterolateral papillary muscle into a cannula used for LV apex decompression. We emphasize the importance of transesophageal echocardiography (TEE) as a valuable tool to identify the underlying cause of low flow in patients undergoing central ECLS with LV apex decompression.

## Case presentation

A 38-year-old female patient with a chief complaint of anorexia received a diagnosis of cardiogenic shock due to COVID-19-related fulminant myocarditis at another hospital. She had no significant medical history. Prompt resuscitation efforts were initiated by concurrently implementing peripheral veno-arterial extracorporeal membrane oxygenation (V-A ECMO) and deploying a pump catheter (Impella CP; Abiomed, Danvers, MA, USA). Organ dysfunction progressed despite percutaneous mechanical circulatory support (MCS), prompting a transfer to our facility for conversion to surgical MCS.

**Fig. 1:**
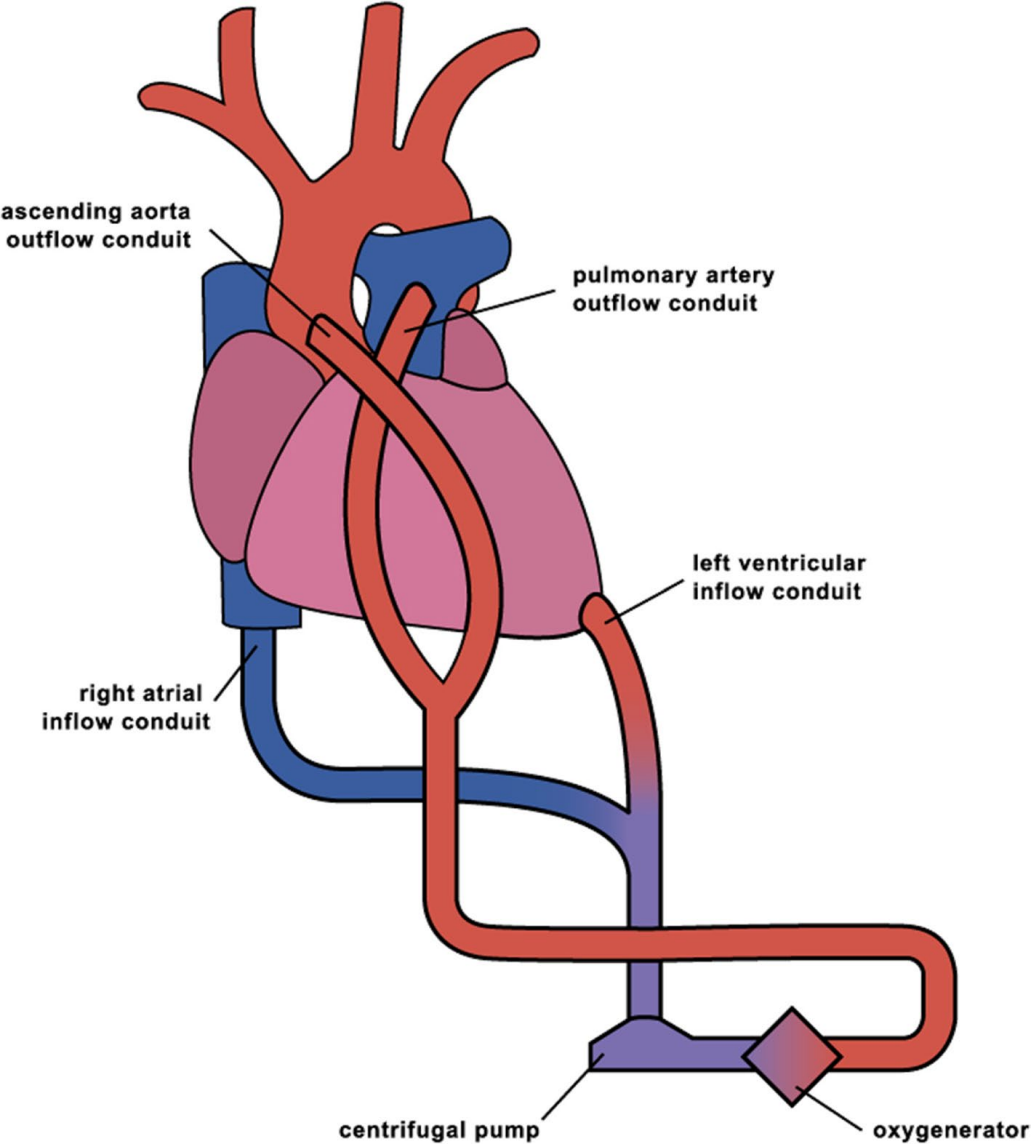
Central extracorporeal life support system with left ventricular apex vent and pulmonary artery return. A central extracorporeal life support (ECLS) system can be established for patients with severe heart failure due to respiratory failure. The process begins with the introduction of a drainage cannula into the left ventricular apex and connection of the return cannula, which goes to the ascending aorta, to a centrifugal pump circuit equipped with an artificial lung. Given that drainage from the left ventricular apex is often insufficient to maintain total flow in such patients, a peripheral venous cannula is inserted via the femoral vein into the superior vena cava–right atrium (SVC-RA) junction. Subsequently, the cannula is connected to a left ventricular drainage cannula using a Y-connector. To prevent the formation of a left ventricular thrombus, another return cannula is placed into the main pulmonary artery when the drainage volume from the left ventricular apex is exceedingly low. The cannula is connected to the ascending aortic return cannula using a Y-connector. The balance of flow between the two cannulas is controlled by using an adjustable clamp and a separate flow sensor located on one of the outflow tubes

We planned a conversion to central ECLS with direct LV decompression via median sternotomy to manage insufficient pump flow, left ventricular distension, and poor oxygenation. In our institution, the location of the outflow and inflow cannula of the central ECLS system is determined by the presence of right heart failure and pulmonary congestion, according to a standardized protocol [3]. Briefly, an extracorporeal LVAD is established by anastomosing an inflow cannula to the LV apex and an outflow cannula to the ascending aorta, using a cardiopulmonary bypass circuit. The flow rate of the extracorporeal LVAD is measured under an appropriate preload of approximately 10 mmHg of right atrial pressure, and if the flow index is less than 2.4, biventricular support is deemed necessary due to right heart failure. Then, an inflow cannula is anastomosed to the right atrium or right ventricle, and an outflow cannula is anastomosed to the pulmonary artery. When severe pulmonary congestion is present and pulmonary vascular resistance is greater than 3 Wood units, blood flow to the pulmonary artery should be limited to less than 3 L/min to avoid exacerbation of pulmonary congestion. In case of high pulmonary vascular resistance, the serial biventricular VAD system could not provide sufficient blood flow. Therefore, a Y-shaped connector is connected to the outflow cannula so that blood can be delivered to both the pulmonary artery and the ascending aorta. In the present case, an outflow cannula was inserted into the partially clamped ascending aorta. An inflow cannula was inserted through a drainage cuff secured to the apex of the left ventricle. Both inflow and outflow cannulae were connected to a centrifugal pump circuit (BIOFLOAT NCVC; Nipro Corporation, Osaka, Japan) integrated with an artificial lung. The initial pump flow was less than 2.4 L/min/m^2^. TEE revealed a narrowed left ventricular cavity and an enlarged right ventricular cavity, indicating that poor drainage from the left ventricular apex was due to right ventricular failure. In patients with pronounced pulmonary congestion, the implementation of a bi-ventricular assist device (BiVAD) system for right heart support carries the risk of exacerbating pulmonary edema and hemorrhage. Our institution therefore uses a variation of the BiVAD system depicted in Fig. 1 for cases with pulmonary vascular resistance (PVR) exceeding 3.0 Wood units. In this patient, the existing inflow cannula placed in the femoral vein was repurposed as an RA (right atrium) inflow cannula, and a second outflow cannula was positioned in the main pulmonary artery. This system, with the drainage and outflow cannulas connected in a Y formation, enables not only the complete unloading of both ventricles but also the regulation of pulmonary blood flow based on the severity of pulmonary edema. This adjustment led to a stable pump flow, with the centrifugal pump set at 5530 rpm, achieving a total flow rate of 8.0 L/min. Based on empirical evidence, the total flow was divided, directing 3.5 L/min (2.15 L/min/m^2^) to the pulmonary artery and 4.5 L/min (2.77 L/min/m^2^) to the ascending aorta. TEE showed a normalized balance between the ventricles, correct orientation of the LV apical drainage cuff, and no suction events. Postoperatively, we managed the patient with deep sedation and mechanical ventilation in the intensive care unit (ICU), aiming to reduce excess extracellular fluid to improve pulmonary congestion and oxygenation.

On the second postoperative day in the ICU, the pump flow intermittently decreased, triggering frequent low-flow alerts. At the time, the pump parameters were set at 5440 rpm, and the systemic flow was 2.3 L/min/m^2^. The external cannula showed no abnormalities such as torsion. The patient remained deeply sedated, showing no signs of asynchrony with the ventilator. An ECG revealed normal sinus rhythm with a heart rate of 105 bpm. The mean arterial pressure, pulse pressure, mean pulmonary arterial pressure, and central venous pressure (CVP) were 65, 5, 19, and 18 mmHg, respectively. Based on these observations, we suspected an abnormal placement of the inflow/outflow cannula. Although transthoracic echocardiography was performed, it failed to produce sufficient images to facilitate a diagnosis, and therefore, the cause could not be determined. Prompt bedside transesophageal echocardiography (TEE) revealed that the anterolateral papillary muscle was suctioned to the vent cannula at the LV apex during systole (Additional file 1: Video 1-pre). This suction was presumed to be a consequence of a reduction in the size of the left ventricular cavity due to diuresis to improve pulmonary congestion and oxygenation. The patient was transferred to the operating room for repositioning of the LV apex cannula under cardiac arrest. Considering that the PVR was less than 3.0 Wood units at the time of weaning from cardiopulmonary bypass, conversion to a BiVAD with an oxygenator was performed according to the standard protocol of our institution. The centrifugal pumps were set to approximately 3500 rpm on both sides, resulting in a pump flow of 4.9 L/min (2.8 L/min/m^2^). TEE showed the absence of left ventricular apical suctioning (Additional file 1: Video 1-post). Postoperatively, there were no low-flow alarms, and the pump flow remained stable. The patient was weaned off BiVAD 12 days after ICU admission and transferred to another facility 34 days after ICU admission. The patient was finally discharged in a stable condition and without any neurological deficits. She continues to be monitored as an outpatient.

## Discussion

LV decompression is a vital strategy for improving early survival in patients experiencing refractory cardiogenic shock [1, 2]. Low pump flow in patients with LV apex decompression presents a considerable threat to their lives, and identifying the underlying cause can be difficult. In the present case, TEE revealed mechanical interference of the anterolateral papillary muscle by an LV apex decompression cannula. This case emphasizes the value of TEE as a vital tool for identifying the cause of low flow.

TEE is a vital tool in diagnosing and managing unstable circulatory states within the ICU [4]. Its ability to detect anatomical and hemodynamic changes has led to its expanded use in the ICU management of various MCS systems, including LVAD [5] and V-A ECMO [6]. While TTE is the preferred initial approach due to its ease of use, availability, and non-invasiveness, it often encounters limitations in postoperative patients affected by factors such as swelling, fluid retention, pulmonary edema, drainage tubes, and MCS devices. Our case highlights the ability of TEE to reveal mechanical interference between the anterolateral papillary muscle and the LV apex decompression cannula, a detail that is challenging to discern with TTE. This demonstrates the essential role of TEE in providing detailed visualization of cardiac structures that are difficult to evaluate with TTE, thereby playing an indispensable role in identifying the causes of low flow.

Addressing low-flow alarms in MCS systems requires a thorough evaluation of the patient. Algorithm-based approaches have been suggested for the management of inadequate drainage in peripheral V-A ECMO [7]. A case report highlighted papillary muscle suction as a cause of low flow in LVAD, advocating for a systematic approach that utilizes both pressure monitoring waveforms and TTE to uncover the root cause of the low flow [8]. However, there is no standardized approach for patients undergoing central ECLS with LV apex decompression. Our case, characterized by severe biventricular failure and a complex central ECLS system, presented circulatory dynamics that were fundamentally different from those in patients with preserved right heart function in LVAD systems or those managed with peripheral V-A ECMO. Therefore, applying existing systematic approaches directly to this case was not feasible.

In our case, the most plausible sequence of events suggests that diuresis, aimed at relieving pulmonary congestion and improving oxygenation, led to a decrease in circulating blood volume, causing a reduction in the size of the LV cavity. As a result, the anterolateral papillary muscle was intermittently suctioned into the LV apical cuff, triggering low-flow alarms. This phenomenon is associated with several key factors: diffuse thickening of the left ventricular wall due to fulminant myocarditis, the occurrence of low flow alarms not immediately after LV apical cuff placement but after initiation of diuresis, and pressure monitoring findings indicating a left heart system problem as evidenced by sudden increases in PAP and CVP. Together, these factors provide a comprehensive explanation for the observed low-flow event.

TEE has demonstrated its usefulness as a diagnostic tool, enabling comprehensive assessment of treatment effectiveness and guiding crucial decision-making processes. This case emphasizes the crucial role of TEE in uncovering the underlying cause of low flow in patients undergoing ECLS with LV apex decompression.

### Supplementary Information

**Additional file 1: Video 1.** Transesophageal echocardiography—transgastric mid-papillary short axis view. Pre: View of the anterolateral papillary muscle suctioned toward the left ventricular apex drainage cuff during systole. Post: View after surgical intervention and repositioning of the left ventricular apex drainage cuff.

## Data Availability

Data sharing is not applicable to this article as no datasets were generated or analyzed during the current study.
